# Maternal stimulation and early child development in sub-saharan Africa: evidence from Kenya and Zambia

**DOI:** 10.1186/s12889-023-17235-w

**Published:** 2023-12-05

**Authors:** Silas Onyango, Patricia Kitsao-Wekulo, Nelson Langat, Kenneth Okelo, Dawn E. Murdock, Jürg Utzinger, Günther Fink

**Affiliations:** 1 Department of Epidemiology and Public Health, Swiss Tropical and Public Health Institute, Kreuzstrasse 2, Allschwil, CH-4123 Switzerland; 2 University of Basel, Petersplatz, Basel, CH-4051 Switzerland; 3 Human Development Theme, African Population and Health Research Center, Nairobi, 10787-00100 Kenya; 4 Episcopal Relief & Development, 815 Second Avenue, New York, NY 10017 USA

**Keywords:** Maternal stimulation, Child development, Early learning, Responsive caregiving, Motor activities, Cognitive activities, Language activities socio-emotional activities

## Abstract

**Background:**

Despite major improvements in child survival over the past decade, many children in low and middle-income countries (LMICs) remain at risk of not reaching their developmental potential due to malnutrition, poor health, and a lack of stimulation. Maternal engagement and stimulation have been identified as some of the most critical inputs for healthy development of children. However, relatively little evidence exists on the links between maternal stimulation and child development exists in sub-Saharan Africa (SSA). This current paper aims to identify the associations between maternal stimulation and child development in Kenya and Zambia, as well as the activities that are most predictive of developmental outcomes in these settings.

**Methods:**

We conducted a descriptive study using data from a prospective study in Kenya and Zambia. The study included three rounds of data collection. Children were on average 10 months old in round one, 25 months old in round two, and 36 months old in round three. The primary exposure variable of interest was maternal stimulation activities, which we grouped into cognitive, language, motor, and socio-emotional activities. The outcome of interest was child development measured through the Third Edition of the Ages and Stages Questionnaire (ASQ-3). Linear regression models were used to estimate the associations between overall maternal stimulation and domain-specific maternal stimulation and child development across the three rounds of the survey.

**Results:**

Higher maternal stimulation scores were associated with higher ASQ scores (effect size = 0.25; 95% CI: 0.19, 0.31) after adjusting for other confounders. For domain specific and child development (ASQ scores), the largest effect size (ES) was found for language stimulation (ES = 0.15) while weakest associations were found for socio-emotional domain activities (ES= -0.05). Overall maternal stimulation was most strongly associated with gross motor development (ES = 0.21) and the least associated with problem-solving (ES = 0.16).

**Conclusion:**

Our study findings suggest a strong positive link between maternal stimulation activities and children’s developmental outcomes among communities in poor rural settings.

**Trial registration:**

NA (not a clinical trial).

**Supplementary Information:**

The online version contains supplementary material available at 10.1186/s12889-023-17235-w.

## Introduction

Globally, child survival has greatly improved in the past three decades, with under-five mortality declining by 59% between 1990 and 2019 [1]. Despite these large improvements in child survival, over 60% of the children living in sub-Saharan Africa continue to be at risk of not reaching their developmental potential [2, 3]. Poor development in children can result from malnutrition, poor health, poverty, lack of stimulation, limited opportunities for early learning, and unresponsive caregiving [4]. Developmental delays pose greater risks not only to children’s health outcomes but also to human capital, income, and general wellbeing across the life course [5, 6]. Providing children with opportunities for early learning through the creation of a stimulating environment offers primary caregivers a unique opportunity to have lifelong positive impacts on their children [7, 8].

The lifelong impact resulting from early stimulation is directly linked to Sustainable Development Goal (SDG) # 4 which aims at ensuring inclusive and equitable quality education and promoting lifelong learning opportunities for all [9]. Children who acquire early developmental skills have the ability to perform well in school, which in turn improves their adult productivity, thereby reducing poverty, and inequalities. In the first few years of life, mother-child interactions are essential for children’s learning and development [10]. Scaffolding theory states that competencies and higher mental abilities develop through interactions and collaborations between children and their caregivers [11].

The interactions and collaborations between the child and their caregivers – often referred to as “maternal stimulation” activities - are very diverse and include reading with the child, storytelling, singing songs, taking the child outside the home for a walk, playing with the child, telling the child the names of objects, and drawing objects with the child. These very basic activities can improve children’s psychological well-being [12], early language acquisition [13, 14], the development of executive functioning [15], socio-emotional skills, and boost the early acquisition of fine and gross motor skills [16]. Previous literature has also linked maternal stimulation to skills that are essential for children’s later development and learning outcomes [17].

A large body of literature has demonstrated the importance of maternal stimulation in high-income settings [18, 19]. In SSA countries, activities such as maternal book reading, storytelling, and naming of objects have been found to be associated with children’s ability to read simple words, latter recognition, and symbol identification [20, 21]. Most of these activities are mainly from urban areas. However, relatively little evidence associating maternal stimulation activities with child development exists in rural settings in SSA countries. The study reported in the current paper aimed to fill this gap using a prospective data set from rural Kenya and Zambia. We aimed to first identify the general associations between maternal stimulation and early childhood development, and then to identify the activities that show the strongest associations with developmental outcomes in rural settings.

## Materials and methods

### Study design

This was a multiple cross-sectional study that utilized secondary data from a community-led parenting empowerment intervention that was implemented in Kenya and Zambia across three survey rounds [22]. The intervention program aimed at improving children’s cognitive, language, motor, social, and emotional development and promoting positive discipline and parenting more generally. The program also empowered primary caregivers (mothers and others in that role) to improve responsive care, early learning, and the security and safety of their children from birth to age three. Trained ECD promoters facilitated parenting social and behavioral change and peer learning with primary caregivers through support and learning groups and ECD home visits. The program was implemented in coordination with local health system staff; the project team complemented and reinforced the health and nutrition work completed by local health volunteers and health staff.

### Study sites

The intervention was implemented by the Episcopal Relief & Development (ERD) team together with the Zambia Anglican Council Programmes (ZACOP) in Zambia and with ACK Development Services (ADS) Nyanza in Kenya. In **Kenya**, data were collected in Nyando sub-County, Kisumu County, specifically in Ayucha, Border 1, and Wanganga sub-locations in Onjiko-Awasi Ward. Kisumu is located in western Kenya, along the shores of Lake Victoria. According to the 2019 census, Kisumu County had a population of about 1,155,574 [23], including 202,519 children under the age of five. The infant mortality rate in Kisumu County was estimated at 54 per 1000 live births. More than half of pregnant women (54%) deliver at home, although attendance at antenatal care services is relatively high, with an estimated 71% of women attending at least three times. The proportion of women using modern contraceptives remains low at 27%, compared with the national average of 46% [24].

Nyando sub-County has some of the poorest health and development indicators in Kisumu County. The sub-County has lower than average uptake of immunization (76.6%, compared to the county average of 82%) and a lower proportion of mothers who had deliveries assisted by skilled attendants (59.4% compared to 70.4% in the county). The proportion of mothers who attended four ANC visits is about 48.4% compared to 49.7% in the county. In addition, the greater Nyanza region has an HIV prevalence rate of 19.3% against a national average of 5.9% [25] which has greatly affected the County.

In **Zambia**, the study was conducted in Mwantaya and Chamuka Wards, which are located in Chisamba District in Zambia’s Central Province. The population in Chisamba District was 103,983 in 2010 [26]. The HIV prevalence rate of 13.4% in Central Province is higher than the national rate in rural Zambia (9.1%). Malnutrition rates are extremely high, with 42.1% of children under five exhibiting stunted growth. Only 46.5% of mothers had deliveries by skilled attendants and less than a quarter (14.4%) of the population has no formal education [27]. According to 2010 data, the total population in the greater Chamuka area was 21,210, with 10,685 males and 10,525 females within 3833 households. Mwantaya Ward is sparsely populated, with little infrastructure and only one health clinic [27].

### Sample size calculation

The sample size was determined following Hemming et al. [4]’s paper on sample size calculation. The number of clusters per arm was fixed at six (three clusters each from the two sub-locations within the intervention sites, and six clusters from a third sub-location in the control arm.). We assumed a minimum detectable effect size of 0.4 with an intracluster correlation (ICC) of 0.03. We also assumed a confidence interval of 95%, a margin-of-error of 5% and 80% power. Hence, the estimated sample size without clustering of data was obtained by the following formula:

$${n=2\left(\frac{{Z}_{1-\raisebox{1ex}{$\propto $}\!\left/ \!\raisebox{-1ex}{$2$}\right.}+{Z}_{1-\beta }}{ES}\right)}^{2}$$

Where.

$${Z}_{1-\raisebox{1ex}{$\propto $}\!\left/ \!\raisebox{-1ex}{$2$}\right.}$$is the critical value of the standard normal distribution corresponding to $$\propto$$ level of signifiance in a two-sided test. Setting $$\propto =5\%$$, then $${Z}_{1-\raisebox{1ex}{$\propto $}\!\left/ \!\raisebox{-1ex}{$2$}\right.}$$= 1.96

${Z}_{1-\beta }$ is the critical value of the standard normal distribution corresponding to $1-\beta$ (power). Setting power to 80% (i.e. $1-\beta =$ 0.8) then ${Z}_{1-\beta }=$0.84

$ES$ is the minimum detectable effect size. We assume an eff ect size of 0.4

$${n=2\left(\frac{1.96+0.84}{0.4}\right)}^{2}=98$$

By fixing the clusters per arms to $k=6$, the required sample size per arms is given by the following formula:

$${n}_{C}=\frac{nk(1-\rho )}{k-n\rho }=\frac{98\times 6\times (1-0.03)}{6-(128\times 0.03)}=186.39\approx 187$$

In the formula above, a feasibility check must be done so that $k>n\rho$. This condition is satisfied since $6>5.61$

Therefore, this study needed a sample size of at least 187 in each site.

### Study sample

**Fig. 1:**
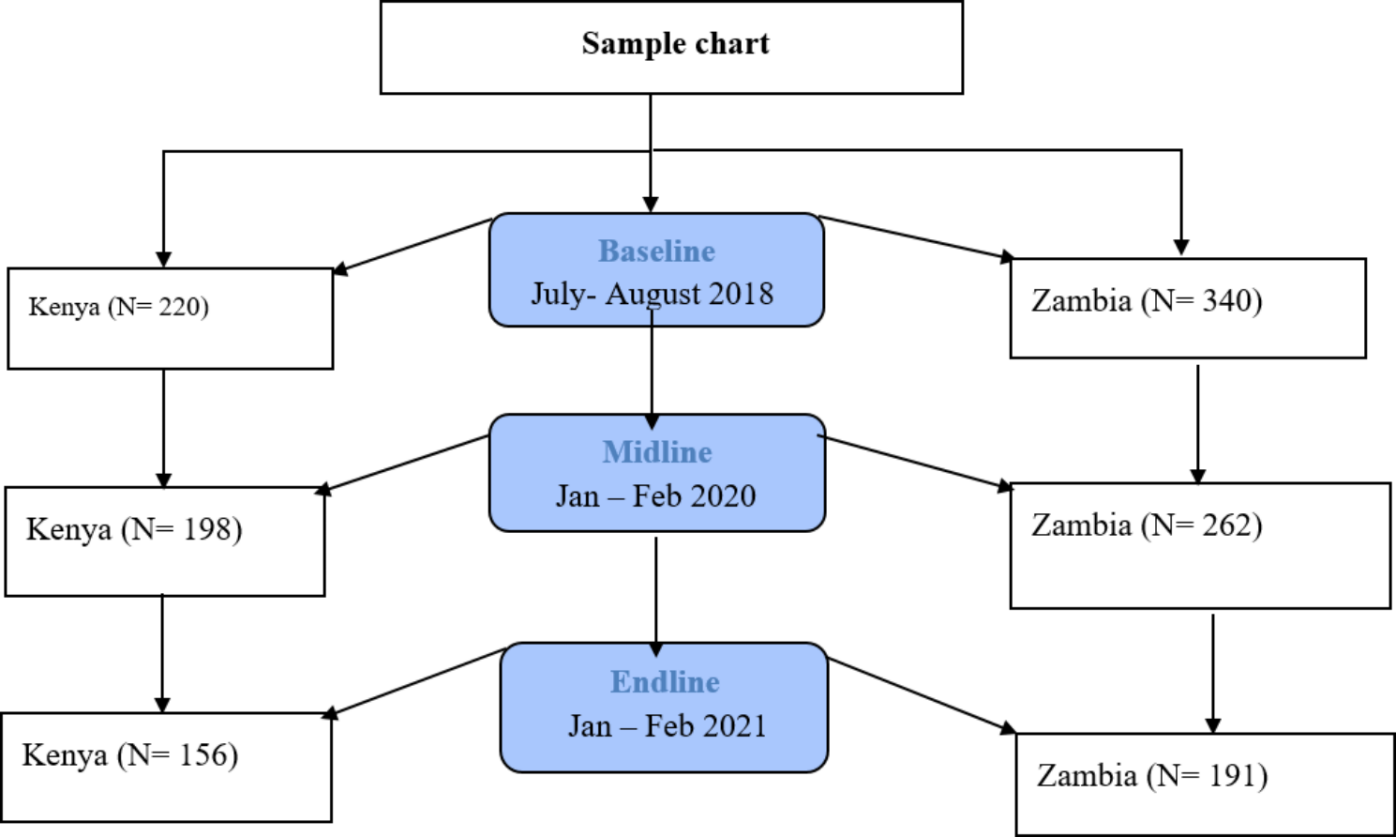
Study sample at each round of data collection by country

In Kenya, 220 mother-infant pairs were recruited across three sub-locations and enrolled in the study during the baseline phase. In Zambia, 340 mother-infant dyads were recruited at baseline across 10 villages within the wards. The study participants were also interviewed at midline after 12 months of intervention implementation and endline after 24 months of implementation (Fig. 1). During the follow-up, 198 and 262 mother-infant dyads were recruited and interviewed in Kenya and Zambia, respectively at the midline data collection while 156 and 191 were recruited and interviewed in Kenya and Zambia, respectively at endline. The caregivers were recruited by the early childhood development (ECD) promoters to participate in the intervention. Since the randomization was done at the village level and the intervention participants were selected based on a given criteria, the sample for the study were conveniently identified and selected from the parent study based on whether they met the criteria such as having a child less than 14 months and also if they consented to participate in the study.

### Exposure and outcome variables

The primary exposure of interest was maternal stimulation. Information on stimulating activities that mothers engaged in with their young children was collected during all three survey rounds. The activities were grouped under four domains based on the primary area of development targeted by the activity: cognitive, language, motor, and socio-emotional activities. Appendix 1 shows the complete list of activities by domain. All the activities were collected through self-reported interviews from the caregivers. Primary caregivers were asked a set of questions to establish their engagement in responsive care and stimulation practices in the previous week that promoted development in specific domains (cognitive, language, motor skills, social and emotional development). Their practices were reported and scored as “Yes = 1” and “No = 0.”

The main outcome variable for the study was child development measured through the Ages and Stages Questionnaire – Third Edition (ASQ-3) [28]. The ASQ-3 relies on caregiver reports and has been validated and used in Zambia [29] as well as in Kenya [30]. The ASQ-3 covers five developmental domains (communication, gross motor, fine motor, problem-solving, and personal-social) as well as an overall development score. The responses to each of the six questions in each domain are summed to provide a score for each area and adjusted to the age of the child. Scores for each domain should fall between 0 and 60. Higher scores indicate outcomes that are more positive for children. Previous study suggest a good interrater and test-retest reliability of 0.94, Cronbach’s alpha coefficient of 0.82 and overall sensitivity and specificity of 0.82 and 0.92 respectively for this tool with variations in the specifics domains and ages of assessments [31, 32]. It also has been validated, translated and used in Kenya, Zambia, Turkey and Colombia to assess children developmental outcomes [33, 34]. The ASQ-3 was administered by trained field interviewers where all responses were recorded electronically using tablets.

### Covariates

To reduce the risk of confounding, we controlled for caregiver age (in years), caregiver employment status, marital status, number of children, family wealth index, and caregivers’ education status in our models. These covariates were included based on the existing literature. Caregiver employment status was conceptualized as either currently employed (including self-employment or small-scale business) or not employed. Marital status was categorized as married or not (divorced, single, or widowed). The number of children consisted of all the children the caregiver lived with, including non-biological children, apart from the index child. Caregiver education was categorized as no education (caregivers who reported no education), primary education (including those with less than 8 years of education), secondary education and above (including college and vocational training), and above secondary education (college and vocational education). To divide households into wealth quintiles, we used principal components analysis. The specific assets included in the principal component analysis were household ownership of a radio, a cellphone, a bicycle, a motorbike, a television, a flush toilet, a fridge, as well as access to piped water.

The predicted value of the first principal component was then used to divide households into wealth quintiles. Further, we included caregiver stress level as measured by the parental stress index (PSI) [35]. In addition, we included information on children’s characteristics such as gender, which was obtained during the interview with the mother. Information on the covariates was collected at the same time that the ASQ-3 questionnaire was administered. We only considered covariates that have been linked to maternal stimulation and child development in the existing literature [17, 36].

### Statistical analysis

We began our analysis with basic descriptive statistics of the study population. Continuous variables were presented using means and SDs while categorical variables were summarized as frequencies and percentages. Second, we performed simple linear regression analyses to estimate the unconditional associations between child development (ASQ scores) and overall maternal stimulation as well as domain-specific maternal stimulation in each round and overall. In addition, linear regression analyses were also performed to estimate the unconditional association between overall maternal stimulation and each of the ASQ domains. Third, we performed multiple linear regression including all the aforementioned covariates as well as controlling for country fixed effects and intervention arm. We used standardized scores for the main exposure and outcome variables in all the models. We reported effect size together with the corresponding p-value and 95% confidence interval. Data analysis was performed using STATA version 16.0 for Windows (STATA Corporation, College Station, TX) [37].

## Results

Descriptive statistics at the baseline of the full sample are presented in Table 1a. Respondents in both countries were aged above 25 years on average. Less than 10% did not have any education. 34% of the women in Kenya and 50% in Zambia were employed. Similar proportions of women across both countries reported that they were married – 80% in Kenya and 70% in Zambia.

**Table 1a:** Descriptive statistics at baseline

	Kenya (N = 220)	Zambia (N = 340)
Child/Caregiver’s characteristics		
Caregiver age (years), mean (SD)	26.7 (8.7)	27.4 (8.3)
**Caregiver education**, N (%)		
No education	4 (1.8)	33 (9.7)
Primary Education	160 (72.7)	214 (62.9)
Secondary and above	56 (25.5)	93 (27.4)
Caregiver employed, N (%)	76 (34.6)	175 (51.5)
Caregiver married, N (%)	184 (83.6)	245 (71.4)
Wealth status, N (%)		
Wealth quintile 1	55 (25.0)	88 (25.9)
Wealth quintile 2	63 (28.6)	75 (22.1)
Wealth quintile 3	28 (12.7)	76 (22.4)
Wealth quintile 4	41 (18.6)	57 (16.8)
Wealth quintile 5	33 (15.0)	44 (12.9)
Parental stress index, mean (SD)	8.3 (7.5)	7.4 (4.5)
Number of children, mean (SD)	3.2 (1.7)	3.1 (2.1)
Child female, N (%)	114 (51.8)	170 (50.0)

Table 1b shows overall trends in exposure and outcome variables per round of data collection. The ASQ mean scores in Kenya increased from 37.5 at baseline to 47.6 at midline and then dropped to 46.9 at endline. In Zambia, the ASQ mean scores increased by 11.7 points, from 37.0 at baseline, to 48.7 at endline. Overall mean stimulation activities increased substantially from 0.48 at baseline to 0.88 at endline. Motor activities showed the greatest improvement from 0.22 at baseline to 0.88 at endline. The progression of the maternal stimulation activities is further presented in Appendix 2. The trend looks similar across all rounds for the overall stimulation and all domain-specific stimulation activities per country with major changes seen between rounds one and two. Appendix 3 shows the comparison of characteristics of participants who completed all surveys versus those who did not complete the surveys.

**Table 1b:** ASQ mean scores and maternal stimulation activities per round

Maternal stimulation activities, mean (SD)		Kenya	Zambia
Round	Variables		
Baseline	ASQ mean scores, mean (SD)	37.5 (20.3)	37.0 (18.1)
	**Developmental Domain**	**Proportion of activities completed**	
	Cognitive development	0.64	0.63
	Language skills	0.37	0.48
	Motor skills	0.22	0.43
	Socioemotional skills	0.68	0.67
	Overall development	0.48	0.56
Midline	ASQ mean scores, mean (SD)	47.6 (11.2)	47.3 (10.8)
	**Stimulation Domain**	**Proportion of activities completed**	
	Cognitive stimulation	0.87	0.80
	Language stimulation	0.70	0.72
	Motor stimulation	0.83	0.80
	Socioemotional stimulation	0.84	0.85
	Overall stimulation	0.81	0.80
Endline	ASQ mean scores, mean (SD)	46.9 (10.1)	48.7
	**Stimulation Domain**	**Proportion of activities completed**	
	Cognitive stimulation	0.93	0.83
	Language stimulation	0.78	0.78
	Motor stimulation	0.88	0.83
	Socioemotional stimulation	0.92	0.85
	Overall stimulation	0.88	0.82

ASQ scores are presented in terms of means of the total sum scores for each of the individual ASQ domains. Overall stimulation scores represent the mean sum of the total of each of the domain-specific stimulation scores across the three rounds. Each of the domain-specific activities is presented in terms of means

Table 2 presents information on both unadjusted and adjusted associations between overall maternal stimulation activities and children’s ASQ z-scores. In the unadjusted model, there was a significant positive association between maternal stimulation score and ASQ score, that is, for every one standard deviation (SD) increase in the mean maternal stimulation score, there is a corresponding 0.25 SD increase in the ASQ score (effect size = 0.25; 95% CI: 0.19, 0.32). After controlling for the other factors in the model, the result remained unchanged. This implies that higher maternal stimulation scores were associated with higher ASQ scores.

**Table 2:** Unadjusted and adjusted associations between overall maternal stimulation and overall ASQ score

Dependent variable: ASQ Zscore	Unadjusted		Adjusted	
	Effect size	95% CI	Effect size	95% CI
Overall maternal stimulation score	0.25^***^	[0.19, 0.32]	0.25^***^	[0.19, 0.31]
**Country**				
Kenya			Reference	
Zambia			-0.08	[-0.27, 0.12]
**Study arm**				
Control			Reference	[0.00, 0.00]
Intervention			0.03	[-0.08, 0.14]
**Survey round**				
Baseline			Reference	
Midline			-0.08	[-0.22, 0.05]
Endline			-0.06	[-0.21, 0.08]
**Caregiver highest education**				
None			Reference	
Primary			-0.09	
Secondary+			-0.05	[-0.32, 0.22]
Caregiver age (years)			-0.00	[-0.01, 0.01]
**Caregiver employment status**				
Employed			Reference	
Unemployed			0.09	[-0.03, 0.21]
**Marital status**				
Married			Reference	
Not married			0.05	[-0.10, 0.20]
Number of children in household			0.02	[-0.02, 0.05]
Caregiver PSI score			-0.00	[-0.03, 0.02]
**Wealth quintile**				
1 (poorest)			Reference	
2			0.06	[-0.11, 0.23]
3			-0.07	[-0.25, 0.10]
4			0.03	[-0.14, 0.21]
5 (Richest)			0.15	[-0.03, 0.33]
Intercept	0.10	[-0.01, 0.21]	0.13	[-0.26, 0.52]
Observations	1366		1366	

^*^p < 0.05, ^**^p < 0.01, ^***^p < 0.001; 95% confidence intervals in square brackets; In the unadjusted analysis, we controlled for country, study arm, and survey round

Table 3 presents the unadjusted and adjusted associations between domain-specific maternal stimulation and child development. Out of the four maternal stimulation domains, three were significantly positively associated with child development in both the adjusted and unadjusted analyses. In the adjusted analysis, the largest effect size (ES) was in the language stimulation (ES = 0.15) while the least was in the socio-emotional domain (ES= -0.05). Adjusting for the other maternal stimulation domains and confounders, for every one SD increase in the language stimulation mean score, the mean ASQ score increased by 0.15 SD (ES = 0.15; CI: 0.07, 0.24). For every one SD increase in the cognitive stimulation mean score, there was a corresponding 0.10 SD increase in the mean ASQ score after controlling for the other maternal stimulation domains and confounders (ES = 0.10; CI: 0.01, 0.18). One SD increase in motor stimulation mean score was associated with 0.09 SD increase in the mean ASQ score after adjusting for the other maternal stimulation domains and confounders (ES = 0.09; CI: 0.01, 0.18).

**Table 3:** Association between domain-specific maternal stimulation and child development

Dependent variable: ASQ Zscore	Unadjusted analysis		Adjusted analysis	
	Effect size	95% CI	Effect size	95% CI
Maternal stimulation domain				
Cognitive stimulation score	0.09^*^	[0.01, 0.18]	0.10^*^	[0.01, 0.18]
Language stimulation score	0.15^***^	[0.07, 0.24]	0.15^***^	[0.07, 0.24]
Socioemotional stimulation score	-0.04	[-0.13, 0.05]	-0.05	[-0.14, 0.04]
Motor stimulation score	0.09^*^	[0.00, 0.18]	0.09^*^	[0.01, 0.18]
Country				
Kenya			Reference	
Zambia			-0.10	[-0.30, 0.10]
Study arm				
Control			Reference	
Intervention			0.03	[-0.09, 0.14]
Survey round				
Baseline			Reference	
Midline			-0.10	[-0.23, 0.03]
Endline			-0.08	[-0.23, 0.07]
Caregiver highest education				
None			Reference	
Primary			-0.10	[-0.34, 0.15]
Secondary+			-0.04	[-0.31, 0.22]
Caregiver age (years)			-0.00	[-0.01, 0.00]
Caregiver employment status				
Employed			Reference	
Unemployed			0.10	[-0.02, 0.22]
Marital status				
Married			Reference	
Not married			0.06	[-0.09, 0.21]
Number of children in household			0.02	[-0.02, 0.05]
Caregiver PSI score			-0.00	[-0.02, 0.02]
Wealth quintile				
1 (poorest)			Reference	
2			0.06	[-0.10, 0.23]
3			-0.08	[-0.25, 0.10]
4			0.04	[-0.14, 0.22]
5 (Richest)			0.16	[-0.03, 0.34]
Intercept	0.11^*^	[0.00, 0.23]	0.15	[-0.23, 0.53]
*Observations*	1366		1366	

^*^p < 0.05, ^**^p < 0.01, ^***^p < 0.001; 95% confidence intervals in square brackets; In the unadjusted analysis, we controlled for country, study arm, and survey round

Further in Table 4, we present the association between overall maternal stimulation activities and individual ASQ domains. There were significant positive associations between maternal stimulation scores and each of the five ASQ domain scores maternal stimulation had the highest absolute effect size on the gross motor domain (ES = 0.21) and the least in the problem-solving domain (ES = 0.16). However, there was no statistically significant difference in the effect of maternal stimulation on each of the five domains as shown by the overlapping 95% confidence intervals. Adjusting for confounders in the model, one SD increase in the maternal stimulation score was associated with 0.19 SD, 0.16 SD, 0.17 SD, 0.21 SD, and 0.18 SD increase in the mean score of communication (CI: 0.13, 0.25), problem solving (CI: 0.10, 0.22), fine motor (CI: 0.12, 0.23), gross motor (CI: 0.16, 0.27), and personal social (CI: 0.12, 0.23) domains of ASQ respectively.

**Table 4:** Association between overall maternal stimulation activities and individual ASQ domains

Dependent variables: ASQ domains	Effect size	95% confidence interval
Communication ASQ domain		
Maternal stimulation score	0.19^***^	[0.13, 0.25]
Problem solving ASQ domain		
Maternal stimulation score	0.16^***^	[0.10, 0.22]
Fine motor ASQ domain		
Maternal stimulation score	0.17^***^	[0.12, 0.23]
Gross motor ASQ domain		
Maternal stimulation score	0.21^***^	[0.16, 0.27]
Personal social ASQ domain		
Maternal stimulation score	0.18^***^	[0.12, 0.23]
*Observations*	1366	

^*^p < 0.05, ^**^p < 0.01, ^***^p < 0.001; 95% confidence intervals in brackets; Multivariate linear mixed effects model used (dependent variables were the individual ASQ domain scores, main independent variable was the maternal stimulation score); Model adjusted for caregiver age, caregiver education, caregiver employment, marital status, number of children in household, PSI score, wealth status, country, study arm, and survey round

**Fig. 2:**
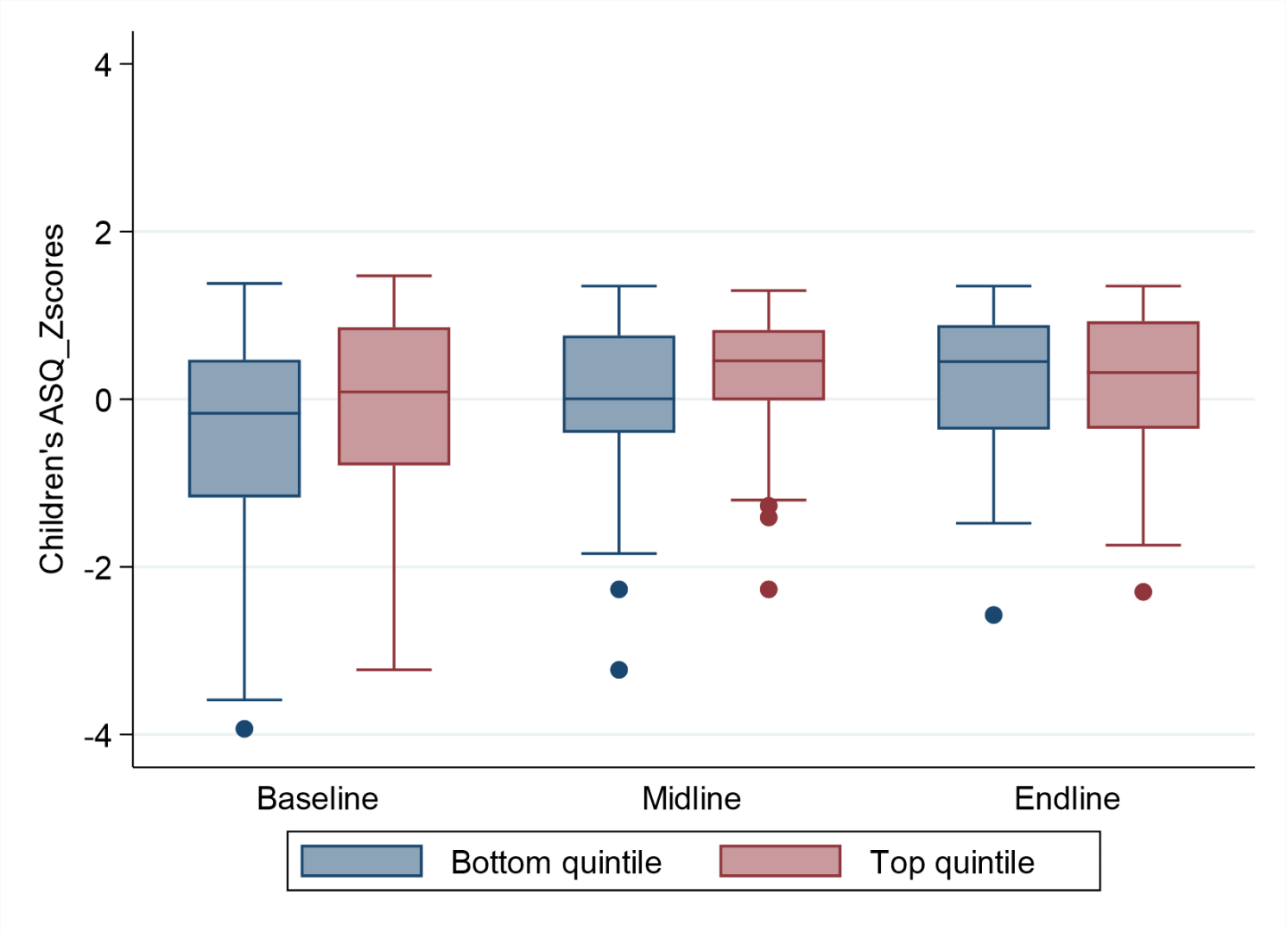
Children’s ASQ scores by round comparing bottom and top wealth quintiles

**Fig. 3:**
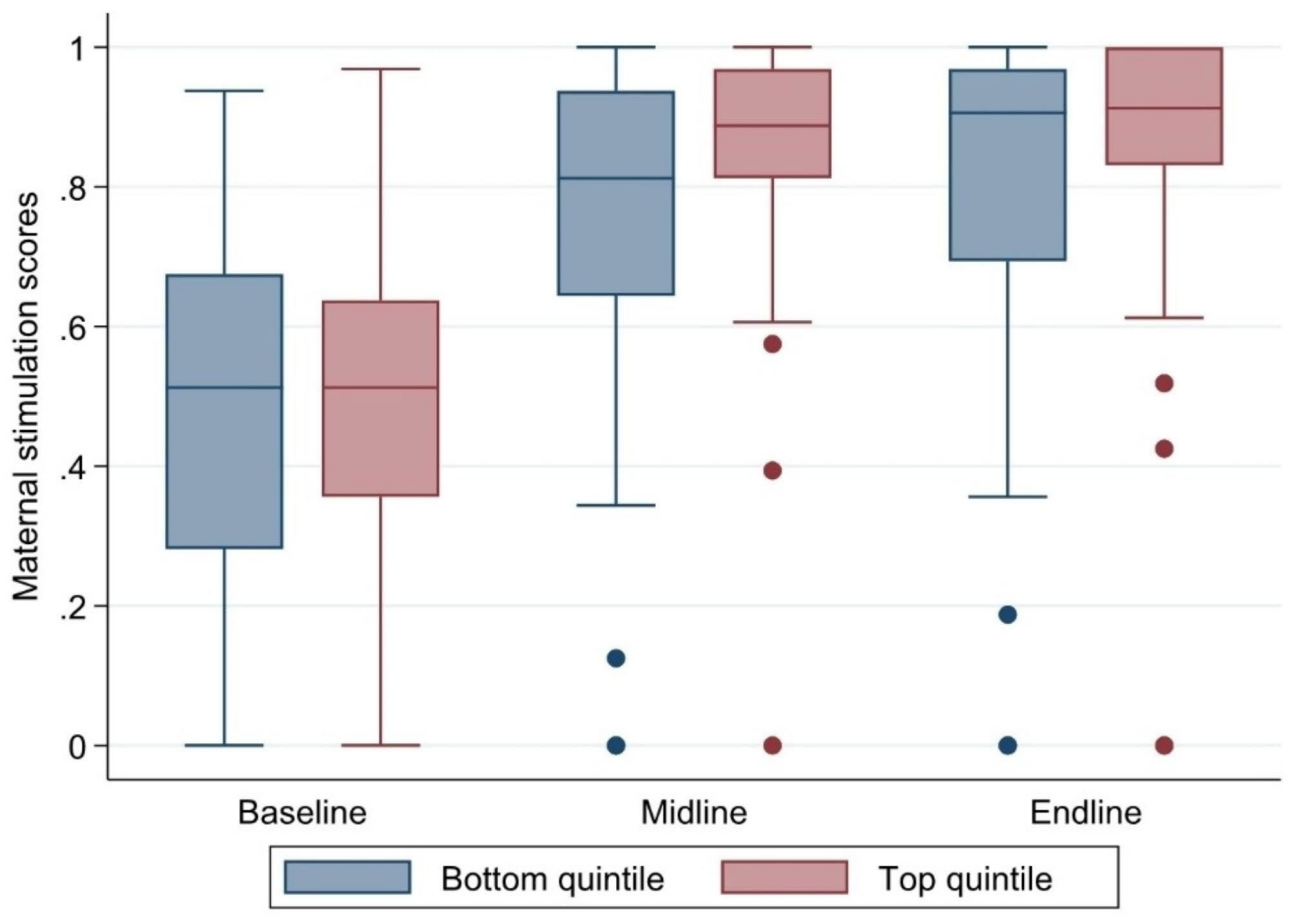
Maternal stimulation scores by round comparing bottom and top wealth quintiles

Figure 2 further compares standardized ASQ scores across wealth quintiles and rounds. The developmental differences between the bottom and top quintiles were initially relatively small at the baseline and then increased at the midline with the quintiles almost the same at the endline. Maternal stimulation scores across wealth quintile and round are presented in Fig. 3. Overall, differences in average stimulation efforts were remarkably small across wealth quintiles, with no difference at all on average in rounds one and three, and very minor differences in stimulation activities were noticed during assessment at round two.

## Discussion

This study investigated the associations between maternal stimulation activities and the developmental outcomes of children in rural Kenya and Zambia. After controlling for covariates, our results indicate that maternal stimulation activities show on average strong positive associations with the overall development of children in these two countries. Strongest associations were found in the first round, when children were on average only 9 to 10 months old. Results on the relative importance of domain-specific stimulating activities appear more mixed, with cognitive and socioemotional activities being most predictive of developmental outcomes in round one (when the children were less than one year old), language activities being most predictive in round two when the children were about two years, and motor activities being most predictive of outcomes in round three when children were over three years. Overall, this suggests that as children’s repertoire of skills becomes more complex and increasingly distinguishable, different stimulation activities may impact development in certain domains more than others [38, 39].

Consistent with other studies, our findings suggest that exposing children to early stimulation activities such as reading, talking, and singing have the potential to improve the acquisition of language and cognitive skills [40], which could contribute to their school readiness and later academic achievement. Acquisition of cognitive abilities, which is one of the most important capabilities of children [41], also appears to be closely linked to stimulation activities. Our finding on the strong link between cognitive development and maternal stimulation is also consistent with previous evidence [19]. Early exposure to telling stories, talking, playing, or naming objects improves children’s cognitive abilities and can prepare them for later academic challenges. Previous studies have also linked the acquisition of gross and fine motor skills to home stimulation [16]. We find that maternal activities such as a hug, kiss, or speaking warmly to the child can also be linked to children’s social and emotional capabilities. These findings are supported by previous research that has shown that children acquire social and emotional skills when they are exposed to a warm and responsive environment [42]. The results of our study suggest that increasing caregiver-child interactions could be an effective tool to enhance the development of children living in disadvantaged settings such as rural areas. Policymakers and program implementers should therefore focus on the parenting programs that aim to improve caregivers’ stimulation knowledge and practices to best support children’s developmental outcomes.

The study had several limitations. While we were able to follow children’s development over time, our measures of child development exclusively relied on the primary caregiver’s self-report and thus were subject to social desirability and reporting bias. In addition, there were a limited number of activities in each of the domains studied, which limited our ability to estimate domain-specific activity effects. There was also a fair amount of attrition over time, which reduced the power of this study. Finally, our cross-sectional may be subject to confounding despite the extensive number of covariates included this is in addition to omitting some important caregiver and child characteristics such as unwanted pregnancy, child health, birth order and birth weight. Overall, this study shows rather large positive short-term associations between caregiver-child interactions and child development. Long-term follow-up studies are needed to understand the long-term effect of this behavior on the later schooling trajectories, academic achievements, and labor outcomes.

## Conclusion

This study suggests large positive associations between maternal stimulation activities and children’s developmental outcomes in poor rural settings. Many of the activities relevant for children under age 3 are relatively easy to carry out, require only minimal materials, and do not require too much time from caregivers. Programs encouraging similar caregiver-child interactions may provide children with the nurturing care and stimulation they need in early life.

### Electronic supplementary material

Below is the link to the electronic supplementary material.

**Supplementary Material 1: Appendix 1**. Maternal stimulation activities. **Appendix 2**. Maternal stimulation activities by round and country. **Appendix 3**. Comparison of characteristics of participants who completed all surveys vs those who did not.

## Data Availability

The datasets generated and/or analysed during the current study are not publicly available due to two-year embargo upon in relation to APHRC policy, but are available from the corresponding author on reasonable request.

## Declarations

Committee for Kenya. In addition, permission to conduct the study was obtained from the National Commission for Science, Technology, and Innovation (NACOSTI) and the Kisumu County Health Management Team before data collection. In Zambia, ethics approval was obtained from the ERES Converge institutional review board (IRB) and also from the Ministry of Health. Written informed consent was sought and obtained from study participants before any data were collected. Informed consent was also obtained from the parents for the children who were under age. For respondents who could not read and write, a thumbprint was taken as a signature but in addition, their husbands or ECD promoters provided informed consent. Informed consent document including a thumbprint signature for those could not read and write were approved by the Amref Health Africa’s ESRC. Confidentiality of the data and the participants’ privacy were respected at all times. Consenting was done at every round of data collection. Consent documents and interview guides were translated into Kiswahili (the national language) and Dholuo (the local language) while in Zambia, the documents were translated into four main Zambian languages (Bemba, Nyanja, Lozi, and Tonga). All methods were carried out in accordance with relevant guidelines and regulations.
