# Supplementary material for: Maternal stimulation and early child development in sub-saharan Africa: evidence from Kenya and Zambia
Source: BMC Public Health. 2023 Dec 5;23:2418. doi: 10.1186/s12889-023-17235-w (PMC10696819; doi:10.1186/s12889-023-17235-w)
Supplement: Supplementary file 1 — Supplementary Material 1: Appendix 1. Maternal stimulation activities. Appendix 2. Maternal stimulation activities by round and country. Appendix 3. Comparison of characteristics of participants who completed all surveys vs those who did not. [file 12889_2023_17235_MOESM1_ESM.docx]

**Appendix 1: Maternal stimulation activities**

| Developmental Areas | | Activities with Children |
| --- | --- | --- |
| COGNITIVE  (Intellectual) | - Provide play materials to the child, including household objects, outdoor objects, toys - Spend time naming things or objects with the child - Play counting games or count with the child - Ask the child questions about what he or she is doing - Take the child outside the compound or yard - Play together | |
| COMMUNICATION (Language) | - Sing with or to the child - Tell stories to the child; encourage the child to tell stories - Look at pictures or read books to the child - Talk to the child while you are doing chores, and/or explain to the child what you are doing - Talk with the child during meals or when feeding the child | |
| MOTOR SKILLS – Fine and Gross) | - Provide small objects for picking up - Provide material for the child to draw with - Encourage the child to run, dance, jump - Encourage the child to kick or throw balls | |
| SOCIAL-EMOTIONAL | - Encourage the child to play with siblings, other children, or family members - Take the child to activities outside the home, such as community or church events - Ask the child to help do simple chores appropriate to their age - Sit with the child during the main meal of the day - Teach about religious or spiritual practices | |
|  | - Hug or kiss the child - Pick up and hold the baby or child when she or he is crying - Carry and hold the baby - Speak to the child in a warm, positive tone of voice - Praise the child for her or his accomplishments - Comfort the child after she or he gets hurt or scared - Feed the child or assist him or her to eat | |

**Appendix 2: Maternal stimulation activities by round and country**

**Appendix 3:** Comparison of characteristics of participants who completed all surveys vs those who did not

| **Variables** | **Did not complete all surveys** | **Completed all surveys** | **p-value** |
| --- | --- | --- | --- |
|  | **N=282** | **N=278** |  |
| Study Arm |  |  | 0.073 |
| Control | 142 (47%) | 161 (53%) |  |
| Intervention | 140 (54%) | 117 (46%) |  |
| Country |  |  | <0.001 |
| Kenya | 86 (39%) | 134 (61%) |  |
| Zambia | 196 (58%) | 144 (42%) |  |
| Caregiver age (Median (IQR)) | 25 (21, 30) | 27 (21, 33) | 0.041 |
| Highest education level |  |  | 0.320 |
| None | 20 (54%) | 17 (46%) |  |
| Primary education | 180 (48%) | 194 (52%) |  |
| Secondary and above | 82 (55%) | 67 (45%) |  |
| Employment |  |  | 0.660 |
| Not_employed | 153 (50%) | 156 (50%) |  |
| Employed | 129 (51%) | 122 (49%) |  |
| Marital status |  |  | 0.680 |
| Not_married | 68 (52%) | 63 (48%) |  |
| Married | 214 (50%) | 215 (50%) |  |
| Wealth quintile |  |  | 0.350 |
| 1 (Poorest) | 69 (48%) | 74 (52%) |  |
| 2 | 66 (48%) | 72 (52%) |  |
| 3 | 62 (60%) | 42 (40%) |  |
| 4 | 47 (48%) | 51 (52%) |  |
| 5 (Richest) | 38 (49%) | 39 (51%) |  |
| PSI score (median(IQR)) | 10 (7, 14) | 8 (5, 13) | <0.001 |
| Number of children in household | 2 (1, 3) | 3 (1, 4) | 0.003 |
| Child’s sex |  |  | 0.870 |
| Male | 138 (50%) | 138 (50%) |  |
| Female | 144 (51%) | 140 (49%) |  |
